# Magnetic Nanoparticles for Antibiotics Detection

**DOI:** 10.3390/nano7060119

**Published:** 2017-05-24

**Authors:** Cecilia Cristea, Mihaela Tertis, Ramona Galatus

**Affiliations:** 1 Analytical Chemistry Department, Faculty of Pharmacy, Iuliu Haţieganu University of Medicine and Pharmacy, 4 Pasteur St., 400349 Cluj-Napoca, Romania; mihaela.tertis@umfcluj.ro; 2 Basis of Electronics Department, Faculty of Electronics, Telecommunication and Information Technology, Technical University of Cluj-Napoca, 28 Memorandumului St., 400114 Cluj-Napoca, Romania; ramona.galatus@bel.utcluj.ro

**Keywords:** antibiotics, magnetic nanoparticles, electrochemical and optical sensors, detection

## Abstract

Widespread use of antibiotics has led to pollution of waterways, potentially creating resistance among freshwater bacterial communities. Microorganisms resistant to commonly prescribed antibiotics (superbug) have dramatically increased over the last decades. The presence of antibiotics in waters, in food and beverages in both their un-metabolized and metabolized forms are of interest for humans. This is due to daily exposure in small quantities, that, when accumulated, could lead to development of drug resistance to antibiotics, or multiply the risk of allergic reaction. Conventional analytical methods used to quantify antibiotics are relatively expensive and generally require long analysis time associated with the difficulties to perform field analyses. In this context, electrochemical and optical based sensing devices are of interest, offering great potentials for a broad range of analytical applications. This review will focus on the application of magnetic nanoparticles in the design of different analytical methods, mainly sensors, used for the detection of antibiotics in different matrices (human fluids, the environmental, food and beverages samples).

## 1. Introduction

Each year in the European Union (EU) alone, over 25,000 people die from infections caused by antibiotic-resistant bacteria. Another problem associated with the misuse of antibiotics is the spread of allergic reactions related to antibiotics intake, impairing the use of certain antibiotics for certain patients and threatening their health. If this trend continues, 300 million people worldwide are expected to die prematurely due to antibiotic resistance over the next 35 years. This is the main reason why the World Health Organization (WHO) declared antibiotic resistance a “*major threat to public health*” [1]. Antibiotic resistance not only has direct consequences for human and animal health. Resistance also presents an economic threat, the result of extra healthcare costs, and productivity losses in EU of at least €1.5 billion each year [2,3]. Moreover, antibiotic waste is discharged into local groundwater and rivers from industrial production and hospital use, harming flora and fauna, and spreading antibiotic resistance. As our environment becomes polluted with antibiotics, the runoff from farms and from other sewers exposes an increasing quantity of the world's bacteria to low concentrations of antibiotics, increasing the antibiotic resistance. Resistance is also societal concern, deriving from the way we use antibiotics, involving different problems: antibiotic overuse, infection control, surveillance for resistance, antibiotic use in animals and crops, and environmental contamination with antibiotics.

The 2015 report of the European medicines agency (EMA) about the consumption of antibiotics for veterinary use showed that the most employed classes of antibiotics are the tetracyclines, penicillins, fluoroquinolones and sulphonamides [4].

The occurrence of pharmaceuticals in the environmental and food samples at trace levels (in the range of nanograms to low micrograms per litre) has been widely discussed and published in the literature. The most used conventional methods for the detection of antibiotics are instrumental [5], such as capillary electrophoresis [6,7], gas chromatography [8,9] and liquid chromatography [9,10] coupled with mass spectrometry [11]. However, these methods are expensive and present limitations, such as the need for expensive laboratory instruments, time consuming separation/clean-up methodologies, long analysis time, extensive sample handling with multiple washing steps, use of expensive and polluting solvents and the impossibility to perform field analyses, making necessary the development of new sensors to overcome these limitations [12]. Electrochemistry and optical based methods appear as interesting and low-cost alternative for antibiotics detection. Sensors are fast and could be easily miniaturized to achieve portable devices that can detect low levels of antibiotics with a high sensitivity.

Magnetic nanoparticles (MNPs) are structures commonly constituted of two different components, a magnetic material and a chemical functionalization layer and have a special property: they can be manipulated using magnetic fields. The magnetic material consists either of iron, cobalt and nickel while the chemical functionalization coating (layer) can be chosen depending on the envisaged applications. Having ferrous or ferric oxide as main constituent, MNPs belong to the class of inorganic based particles. This iron oxide based core could be coated by either inorganic materials (silica, gold), or organic materials (phospholipids, fatty acids, polysaccharides, peptides or other surfactants and polymers) [13]. The size of MNPs starts from 5 to 500 nm in diameter in the case of nanoparticles and from 500 nm to 500 µm in diameter in the case of microbeads. Usually under the name magnetic nanobeads, magnetic nanoparticle clusters can be found, a number of individual magnetic nanoparticles with a diameter in between 50 and 200 nm [14].

The special properties of MNPs made them very attractive for research during the last decade because of their potential use in catalysis [15], as contrast agents in magnetic resonance and magnetic particle imaging [16,17], magnetic data storage [18], micro and nanofluidic [19,20] photonic and optical applications [21,22], magnetic inks for jet printing, tissue targeting and biomedicine [23,24], sensors [25,26], biosensors [27,28,29], and environmental applications [30].

The main properties of magnetic particles intensively exploited for medical applications are their biocompatibility, injectability, lack of toxicity, and high-level accumulation in the target tissue or organ [31]. Magnetic particles are attracted to high magnetic flux density. This feature is used for drug targeting and bioseparation including cell separation and purification. Currently, MNPs are used as contrast agents for magnetic resonance imaging and heating mediators for cancer thermotherapy (hyperthermia). Their magnetic properties, based on their inducible magnetization, allow them to be directed to a defined location or heated in the presence of an externally applied magnetic field. Furthermore, a novel application of magnetic nanoparticles as a platform for the immobilization of the biocompound, for immuno/aptasensor development has been proposed.

Already commercial available, so-called beads can be functionalized with molecules that allow a specific adsorption of proteins or other biomolecules, and subsequent separation in a magnetic field gradient for diagnostic purposes. Besides their use in nanomedicine, MNPs have shown promising performances in pollutant detection or removal, with application in membrane separation water treatment and purification processes [32].

Few examples were devoted to critical reviews on sensing using MNPs for pollutants and drug detection from different matrices (body fluids, food, beverages, or environmental samples). In the design of sensors, MNPs were incorporated in order to increase sensitivity, or for multiplexing.

Several electrochemical and optical sensors used for selective and sensitive detection of common classes on antibiotics in environmental samples will be presented, focusing on MNPs synthesis and surface modification.

## 2. Synthesis and Characterization of MNPs

Synthesizing the different coatings of nanoparticles represent a critical step in their further use in in vivo and in vitro applications. By changing the pH, the additive in the solution as in cell media or body fluids, their viscosity and any other fluidic properties, the colloidal stability, and behavior are strongly influenced. In order to be used for biomedical applications and in sensors design, the long term stability, homogeneous and continuous coatings with tailored materials are demanded.

**Figure 1:**
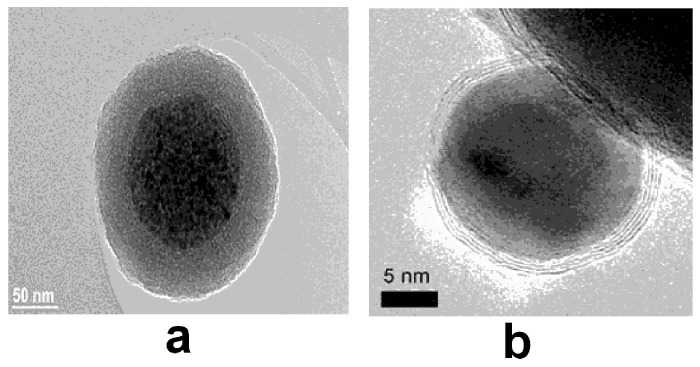
Transmission Electron Microscopy (TEM) images of: (**a**) a maghemite magnetic nanoparticles (MNP) cluster modified with silica shell “Reproduced with permission from [36]. American Chemical Society, 2015”; (**b**) a cobalt nanoparticle with graphene shell “Reproduced with permission from [39]. Wiley—VCH Verlag GmbH & Co., 2007”.

MNPs can be classified according to their composition as:

- Oxides (ferrites), also known as iron oxide nanoparticles, are the most common and widely used MNPs. There are eight iron oxides known, three of them being very popular: hematite (α-Fe_2_O_3_), maghemite (γ-Fe_2_O_3_) and magnetite (Fe_3_O_4_) for specific technical and biomedical applications [33]. If the diameter of the particles is maintained smaller than 128 nm, the self agglomeration of the particles can be avoided while this MNPs exhibits magnetic behavior only in the presence of an external magnetic field. This is due to the supermagnetic properties of these MNPs [34]. The surface of ferrite based nanoparticles can be often modified by different compounds, such as surfactants, silica and other derivatives in order to increase their stability in aqueous media [35]. An example of this type of MNPs is presented in Figure 1a [36].
- Ferrites with a shell, when the surface of MNPs is inert and any functionalization through covalent bounding is not possible in this case. The reactivity of this MNPs can be improved after the surface coverage (coating) with silica that can be easily functionalized with different compounds [37,38].
- Metallic nanoparticles, present only the metallic core, and can be more suitable for biomedical applications due to their higher magnetic moment versus oxides. Important drawbacks include the properties of being pyrophoric, and presence of high reactivity to oxidizing agents.
- Metallic nanoparticles with a shell, consisting of the coverage of metallic core with a shell obtained after gentle oxidation or reaction with surfactants, polymers or precious metals. For example, Co nanoparticles were covered with an anti-ferromagnetic CoO layer formed in the presence of oxygen environment or, one with a shell made of graphene (Figure 1b) [39].

In order to synthesize MNPs with different composition, several oxides (such as Fe_3_O_4_ and Fe_2_O_3_), pure metals (as Fe and Co), spinel-type ferromagnets, such as MgFe_2_O_4_, MnFe_2_O_4_, and CoFe_2_O_4_ or alloys of Co, Fe and Pt (CoPt_3_ and FePt) could be used [40]. Depending on the desired shape, stability, and dispersion of MNPs the synthesis method is chosen accordingly. For obtaining high quality MNPs popular methods include co-precipitation, thermal decomposition and/or reduction, micelle synthesis, flow injection syntheses, micro emulsions, sol-gel synthesis, electrospray synthesis, hydrothermal synthesis, hydrolysis and thermolysis of precursors and laser pyrolysis techniques. The colloidal nature of MNPs is challenging mainly because the synthesis must lead to a monodisperse population of magnetic grains with suitable size, and in a reproducible manner in order to produce large quantities without the need of complex purification procedures.

Several methods of MNPs synthesis were developed and are currently used [41,42]. The most common method for the production of MNPs is the chemical co-precipitation of iron salts [41].

### 2.1. Co-Precipitation

Co-precipitation is a facile and convenient way to synthesize iron oxides (either Fe_3_O_4_ or Fe_2_O_3_) from aqueous Fe^2+^/Fe^3+^ salt solutions by the addition of a base under inert atmosphere at room temperature or at elevated temperature. There are many factors influencing the size, shape and composition. This include the type of the salt used, the ratio between Fe^2+^/Fe^3+^, the pH and ionic strength of the media, and temperature. Once the experimental condition is set, the quality of MNPs obtained with this method is high and reproducible. A new approach using organic additives as stabilization and/or reducing agents allowed the preparation of monodisperse MNPs with different sizes from 2 to 8 nm, after adjusting the molar ratio of citrate ions and metal ions (Fe^2+^ and Fe^3+^). Recent studies showed that oleic acid is the best candidate for the stabilization of Fe_3_O_4_ [43].

### 2.2. Thermal and Hydrothermal Decomposition

Hydrothermal synthesis of Fe_3_O_4_ nanoparticles have been intensively applied in aqueous media and requires autoclaves that can be operated at high pressure and temperatures above 200 °C. The main reactions that take place are hydrolysis and oxidation, and special care must be taken with regard to the reaction conditions (solvent, temperature, and time) that can influence the final product. Under hydrothermal conditions a broad range of nanostructured materials can be formed. This method is based on a general phase transfer and separation mechanism occurring at the interfaces of the liquid, solid, and solution phases present during the synthesis. MNPs with smaller size could be synthesized by using thermal decomposition of organometallic compounds in high boiling organic solvents containing stabilizing surfactants monodisperse, as reported by Sun et al. [44,45,46]. Moreover, Fe_3_O_4_ and CoFe_2_O_4_ nanoparticles can be prepared in very uniform sizes of approximately 9–12 nm, respectively. Li et al. also reported on the synthesis of monodisperse, hydrophilic, single crystalline ferrite microspheres by hydrothermal reduction [47].

### 2.3. Microemulsion and Inverse Micelles

This is another method used for MFe_2_O_4_ synthesis (where M could be Mn, Co, Ni, Cu, Zn, Mg, or Cd, etc.). These types of MNPs are the most important magnetic materials for electronic applications. Spinel ferrites can be synthesized in microemulsions and inverse micelles. For instance, MnFe_2_O_4_ nanoparticles with controllable sizes from 4 to 15 nm were synthesized through the formation of water-in-toluene inverse micelles with sodium dodecylbenzenesulfonate as surfactant as reported by Liu et al. [48]. Different shapes could be prepared by this technique such as spheroids, oblong cross section or tubes. Although many types of MNPs have been synthesized in a controlled manner using the microemulsion method, the particles size and shape usually vary over a relative wide range. Other disadvantages are the quite narrow working window for the synthesis in microemulsions, the yield of nanoparticles (low compared to other methods, such as thermal decomposition and co-precipitation), and the large amounts of necessary solvent. It is thus not a very efficient process and also rather difficult to scale up.

### 2.4. Sol-Gel Processes

This process is a classical wet chemical technique widely used in the fields of materials science and ceramic engineering. Such a method is used primarily for the fabrication of materials (typically metal oxides). Generally, sol particles may interact by Van der Waals forces or hydrogen bonds, and a gel may also form linking polymer chains. In most gel systems used for materials synthesis, the interactions are of a covalent nature and the gel process is irreversible. The gelation process may be reversible if other interactions are involved. Typical precursors for the synthesis of iron oxide nanoaparticles (IONPs) are formed through iron alkoxides and iron salts (such as chlorides, nitrates and acetates), which undergo various forms of hydrolysis and polycondensation reactions. These reactions are performed at room temperature, and further heat treatments are needed to acquire the final crystalline state. By this method, the IONPs will form through at least a two step phase transformation: Fe(OH)_3_ → *β*-FeOOH → *γ*-Fe_2_O_3_ [49]. The final properties of MNPs are highly dependent upon the structure created during the sol stage of the sol-gel process [50].

### 2.5. Biosynthesis

This is a green and ecofriendly method using bacteria’s and other microorganisms capable of reducing or oxidizing salts into MNPs. Recently, new types of bacteria have been employed to synthesize magnetic MNPs. For example, Bharde et al. have reported that the bacterium *Actinobacter* sp. was capable of synthesizing maghemite nanoparticles (NPs) under aerobic conditions when reacted with a ferric chloride precursor. Moreover, maghemite NPs showed superparamagnetic characteristics as expected. Compared to the earlier reports on synthesis of magnetite NPs by magnetotactic bacteria and iron reducing bacteria, which took place strictly under anaerobic conditions, the reported procedure offered a significant advance since the reaction occurred under aerobic conditions [51]. Another approach reported by Sundaram et al. showed the ability of *Bacillus subtilis* strains isolated from rhizosphere soil to produce IONPs. This successful synthesis of stabilized Fe_3_O_4_ NPs, which was capped by organic molecules, indicates the applicability of the isolated *Bacillus subtilis* strain for the bulk synthesis of IONPs [52].

### 2.6. Functionalization of the Surface of NPs

This is a method for tuning the overall properties of particles to fit targeted applications [53]. Biomedical applications often require a strict control of the MNPs interfaces. Beside the need for colloidal stability in a complex biological environment, a major requirement for the successful integration of MNPs in biomedical and sensing application is the minimization of biologically non-specific adsorption events. Such non-specific events can drastically hamper molecular recognition processes at the surface of the MNPs, therefore reducing the efficiency of MNPs based bioassays. Since the MNPs are magnetically attracted to each other, beside the flocculation that occurs due to Van der Waals forces, it is important to functionalize their surface in order to stabilize it. Some examples of such stabilizers are surfactants (sodium oleate, sodium carboxymethylcellulose) and polymer that prevent aggregation. The modifiers for the surface of MNPs are both inorganic and polymeric (organic) materials, the last ones being both synthetic and natural. Examples of synthetic polymers used for MNPs coating are: poly(ethyleneglycol), poly(vinyl alcohol) poly(ethylene-co-vinyl acetate), poly(vinylpyrrolidone), poly(lactic-co-glycolic acid), while chitosan, gelatin, dextran are classified as natural polymers [24]. These methods are summarized in Table 1 highlighting the advantages and disadvantages of each.

## 3. Types of Antibiotics and Their Mechanism of Action

Antibiotics belong to a class of drugs (synthetic, semisynthetic, and natural compounds) capable to treating a wide variety of infectious diseases, and promoting animal growth by improving feed efficiency [56]. Their role in fighting against dangerous bacteria cannot be neglected. Their intensive use for the treatment of meat producing animals generates the risk to human health due to the transmission of the residues and metabolites of these compounds into the food chain [57]. Human beings may be at risk after long time exposure becoming hypersensitive and developing allergic reactions, or more severe health problems. The biggest fear is related to the wide application of antimicrobial agents contributing to the rise and spread of antibiotic resistant bacterial infections, so called bugs. For the record, the EU sales of veterinary antimicrobial agents in 24 countries (excluding Croatia, Greece, Malta, and Romania) were 7974.2 tons of pure ingredients in 2012 as reported by EMA [58]. Several examples of widely used antibiotics for human and veterinary purpose are presented in Table 2.

The most used antibiotics are: tetracyclines, penicillins, sulphonamides, macrolides and fluoroquinolones. These classes of antibiotics are targeted regarding the need for fast and sensitive methods and devices for antibiotics detection in food, beverages, and environmental samples (such as water, waste waters, air, and soil).

### 3.1. Tetracyclines

Tetracyclines are broad spectrum antibiotics known as “derivatives of polycyclic naphthacene carboxamide” derived from cultures of Streptomyces bacteria, used in the treatment of infections of the urinary tract, respiratory tract, intestines, but also used in the treatment of Chlamydia, especially in patients allergic to β-lactams and macrolides. Tetracyclines action is based on the inhibition of protein synthesis after uptake into susceptible organisms, by reversibly binding to the 30S ribosome of the microbial RNA, and preventing the attachment of aminoacyl-tRNA with the acceptor site on the 70S ribosome. This represents a bacteriostatic and not bacteriocidal activity [60]. However, their use for these indications in humans is less popular than it once was due to widespread development of bacterial resistance. They are mainly and extensively used in veterinary and aquaculture medicine, excreted to environment with a combination of intact and metabolized pharmaceuticals. The residues of these antibiotics have been identified in water, soil and other environments [61].

### 3.2. Penicillins

This class of antibiotics was among the first medications to be effective against many bacterial infections caused by staphylococci and streptococci, still used today, even though many types of bacteria have developed resistance. Penicillin structure is based on a four-membered β-lactam ring; this structural moiety being essential for penicillin’s antibacterial activity [62]. Penicillin is actively excreted, and about 80% of a penicillin dose is cleared from the body within three to four hours of administration [63]. The generically used term “penicillin” refers to benzylpenicillin (penicillin G), procaine benzylpenicillin, benzathine benzylpenicillin, and phenoxymethylpenicillin (penicillin V). All these types of penicillin antibiotics have almost the same antibacterial activity, and can still be used to treat a wide range of infections caused by bacteria although the number of penicillin resistant bacteria is increasing [64,65,66].

### 3.3. Sulphonamides

These are a group of synthetic medicines that contain the sulfonamide chemical group. They are also called sulfa drugs (sometimes spelled as sulpha drugs or sulphonamides). Sulfanilamide was the first sulfonamide developed in 1906, although it was not used as an antimicrobial until the late 1930s. Several thousand other substances have since been developed from sulfanilamide [67]. Sulfonamide antimicrobials work by interfering with the synthesis of folic acid in bacteria, which is essential for nucleic acid formation and ultimately DNA and RNA. People obtain folic acid from their diet but bacteria need to synthesize it. When used alone, sulfonamides antibiotics are bacteriostatic (stop bacteria from reproducing but don't necessarily kill them); however, in combination with trimethoprim (co-trimoxazole), which acts at a different enzyme in the pathway of folic acid synthesis, sulfonamides tend to be bactericidal (kill bacteria). Many sulphonamides are rapidly excreted and are very soluble in urine so they are used to treat infections of the urinary tract [66].

### 3.4. Macrolides

Macrolides are antibiotics that are primarily bacteriostatic; by binding to the 50S subunit of the ribosome they inhibit bacterial protein synthesis. Macrolides are active against many aerobic and anaerobic Gram-positive cocci, except for most enterococci, many *Staphylococcus aureus* strains (especially methicillin-resistant strains), and some *Streptococcus pneumoniae* and *Streptococcus pyogenes* strains, *Mycoplasma pneumoniae*, *Chlamydia trachomatis*, *Chlamydophila pneumoniae*, *Legionella* sp., *Corynebacterium diphtheriae*, *Campylobacter* sp., *Treponemapallidum*, *Propionibacterium acnes*, *Borrelia burgdorferi* etc. Macrolides have been considered the drug of choice for treating streptococcal and pneumococcal infections, when penicillin cannot be used. Their mechanism of action relies on the inhibition of bacterial protein biosynthesis, and they are thought to do this by preventing peptidyl transferase from adding the growing peptide attached to RNA to the next amino acid as well as inhibiting ribosomal translation [66,68].

### 3.5. Fluoroquinolones

The quinolones are synthetic broad-spectrum antibiotic drugs [69] that were first isolated from natural sources (such as plants, animals and bacteria) and can act as natural antimicrobials and/or signaling molecules. Quinolones exhibit concentration dependent bactericidal activity by inhibiting the activity of DNA gyrase and topoisomerase, enzymes essential for bacterial DNA replication.

Quinolones are divided into 4 groups, based on antimicrobial spectrum, clinical indication and pharmacology:

- First generation drugs (e.g., nalidixic acid)
- Second generation quinolones (e.g., ciprofloxacin) have increased gram-negative and systemic activity
- Third generation drugs (e.g., levofloxacin) have expanded activity against gram-positive bacteria and atypical pathogens
- Fourth generation quinolone drugs (currently only trovafloxacin) add significant activity against anaerobes

The newer fluoroquinolones have broad-spectrum bactericidal activity, excellent oral bioavailability, good tissue penetration and favorable safety and tolerability profiles [70].

Many newer quinolones including trovafloxacin, gatifloxacin, grepafloxacin, temafloxacin, lomefloxacin, sparfloxacin, and enoxacin have been withdrawn because of severe hepatic toxicity, of hypoglycemia and hyperglycemia, of cardiac toxicity, of acute renal failure, hemolytic anemia and coagulopathy, respectively. Most fluoroquinolones are metabolized in the liver and excreted in urine, thus high levels of these drugs can be found in urine. The percentage of the excreted quinolones under non-metabolized form vary from 19% to 83% [66,71].

## 4. Analytical Methods for Antibiotic Detection and Quantification Based on MNPs

The most conventional methods used to quantify pharmaceuticals in water (drinking, effluents, and waste waters) are gas chromatography with mass spectrometry (GC-MS), tandem mass spectrometry (GC-MS/MS), liquid chromatography with mass spectrometry (LC-MS) or tandem mass spectrometry (LC-MS/MS). These analytical techniques are able to detect target compounds down to the nanogram per liter. Traditional methods for determination of antibiotics include microbiological inhibition tests, immunoassays, and chemical physical methods such as gas/liquid chromatographic (GC/LC) analysis and capillary electrophoresis (CE). Among them, microbiological tests are relatively slow and nonspecific, and immunoassays are usually expensive [61].

**Figure 2:**
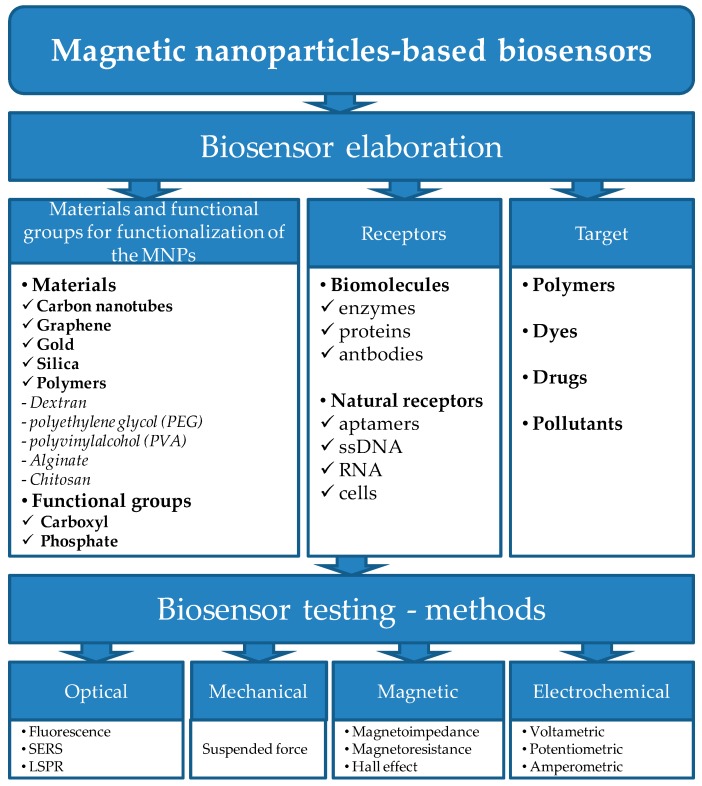
The components for magnetic biosensor elaboration and methods for testing.

Antibiotic biosensing started to be employed in the last decade due to high selectivity through the presence of biomolecules, and high sensibility gained by the use of nanomaterials (see MNPs as depicted in Figure 2).

Other analytical techniques and examples of sensors used for antibiotic detection are summarized in Table 3.

Sample extraction and concentration of antibiotics for high performance liquid chromatography (HPLC) detection is necessary in all cases for separating antibiotics from the background material. A large number of methods such as lyophilization, liquid-liquid extraction and solid phase extraction have been employed. Various improvements for each of these methods has been tested including the use of MNPs and combination of MNPs with carbon nanotubes CNT, graphene, β-cyclodextrines for solid phase extraction (SPE).

However, these efficient methods display some drawbacks such as the necessity of laboratory instruments and skilled technicians, time-consumption as they require separation/clean-up methodologies, long analysis time, extensive sample handling with multiple washing steps, the use of expensive and polluting solvents, and the difficulty to perform rapid field analyses. The development of rapid and low cost multi-target analytical methods able to detect a wide variety of antibiotics is of interest. Among these techniques, electrochemistry based methods appear as a pertinent alternative as they are fast and could be easily miniaturized to achieve portable sensors. In this context, electroanalysis using specific electrodes are the subject of increasing interest as a way to design selective and sensitive sensors. The development of such electrodes requires the immobilization of receptors specific to the antibiotics on the electrode surface.

### 4.1. Electrochemical Sensors for Antibiotics

As defined by the International Union of Pure and Applied Chemistry (IUPAC), “a chemical sensor is a device that transforms a chemical information, ranging from the concentration of a specific sample component to total composition analysis, into an analytically useful signal. The chemical information, mentioned above, may originate from a chemical reaction of the analyte, or from a physical property of the system investigated” [83]. In accordance with transduction method, chemical sensors can be classified as: electrochemical, piezoelectric, optical, thermal and magnetic. When using a bioelement as a recognition element attached to the surface with a transducer, the selectivity of the sensor is much improved.

The first generation of biosensors are the enzyme based sensors, and over the years, a large variety of this type was developed. In enzymatic biosensor, an electrochemical signal (i.e., redox related current) is measured in relation to the conversion of the analyte in an electroactive species by the bioreceptor (i.e., transducer integrated enzymes, cellular organelles, tissues or whole microorganisms). If the transducer incorporates a biological or biomimetic receptor molecules that can reversibly bind the target analyte with high selectivity in a non-destructive way we are referring to affinity biosensors [84]. In that case, the biorecognition molecules include antibodies, nucleic acids, molecular imprinted polymers, peptides and lectins. The major advantage of the affinity biosensors is their ability to distinguish between compounds belonging to the same class of molecules. The most used bioelements are antibodies and aptamers since they could be easily retained at the surface of MNPs. Immunosensors, being a class of biosensors, are analytical devices coupling the immunochemical reaction with a transducer. They belong to the most important class of affinity biosensors which are based on the specific recognition of antigens by antibodies (Ab) to which they form a stable complex that is formed also in immunoassays [85].

Aptamers, first reported in 1990, are oligonucleic acid sequences of 30 to 100 nucleotides that are generally prepared by dynamic combinatorial chemistry from a technique called Systematic Evolution of Ligands by Exponential Enrichment (SELEX). Aptamers are frequently called synthetic Ab since they can specifically recognize various target molecules ranging from small ions to large proteins with high affinity and selectivity. Moreover, compared with Ab, they present some advantages: accurate and reproducible chemical production, stability under a wide range of buffer conditions, resistance to harsh treatments without losing their bioactivity, and the thermal denaturation is reversible for aptamer. Even though the aptamers were initially used as therapeutic agents, their ability as recognition element for biosensing has been observed only recently since the first electrochemical aptasensor was reported in 2004 [86].

The use of magnetic micro- and nanoparticles either as immobilization platforms or as labels has attracted major attention. Their use as a solid support for the immobilization of the recognition element has many advantages such as fast and specific immobilization of a wide amount of bioelements, easy separation after the washing and reaction steps, easy manipulation, reduction of the analysis time and reagents consumption [87].

Many approaches using magnetic bead based biosensors with high sensitivity and high selectivity have been tested and reported, and are bringing new insights into early disease diagnosis and drug analysis [77,78,79]. These biosensors are not only based on MNPs properties but also on their functionalization. Several MNPs based sensors were developed for antibiotics detection. For instance, to detect sulfonamide antibiotic residues in food samples, an affinity based electrochemical immunosensor has been developed based on a graphite composite electrode, biofunctionalized MNPs, and electrochemical nanoprobes prepared by labeling the specific Abs with CdS nanoparticles (CdSNP). After the immunochemical reaction, the CdSNP were dissolved and the metal ions released are reduced at the electrode, and read as in the form of current or charge signal, by the anodic stripping technique. Due to the amplification effect on the amperometric signal produced by the CdSNP, higher detectability can be reached. Sulfapyridine, one of the most widely used sulfonamide congeners, can be detected in honey samples prepared in buffer at a concentration of 0.25 mg L^−1^. The use of MNPs minimizes the matrix effect allowing a limit of detection of 0.11 mg kg^−1^ (current measurements), far below the limits established in some countries for the sulfonamide residues in honey samples [88].

The simultaneous detection of oxytetracycline (OTC) and kanamycin (KAN) was achieved based on metal organic frame materials (MOFs) doped with other metal ions as signal tracers and RecJfexonuclease-catalyzed targets recycling amplification. The aptasensor consists of capture beads (the anti-single-stranded DNA Ab, as anti-ssDNAAb, labeled on Dynabeads) and nanoscale MOF (NMOF) based signal tracers (simplified as Apts-MNM, the NMOF labeled with metal ions and the aptamers). The aptasensor was formed by the specific recognition between anti-ssDNAAb and aptamers. In the presence of both targets, aptamers prefer to form targets-Apts-MNM complexes instead of anti-ssDNAAb-aptamer complexes, which results in the dissociation of Apts-MNM from capture beads. With the employment of RecJfexonuclease, targets-Apts-MNM in supernatant was digested into mononucleotides and liberated the target, which can further participate in the next reaction cycling to produce an increasing number of signal tracers. After magnetic separation, the enhanced square wave voltammetry (SWV) signals were produced from signal tracers. The aptasensor exhibited a linear correlation in the range from 0.5 pM to 50 nM, with detection limits of 0.18 pM and 0.15 pM (S/N = 3) toward OTC and KAN, respectively. This strategy has promising applications in food analysis [89].

Another electrochemical sensor based on polyvinyl pyrrolidone capped CoFe_2_O_4_@CdSe core shell modified electrode for a rapid detection and highly sensitive determination of rifampicin (RIF) by square wave adsorptive stripping voltammetry was reported. RIF, 3-([(4-methyl-1-piperazinyl) imino] methyl) is an important antibiotic drug which is used in the treatment of several serious infectious such as tuberculosis, leprosy and HIV. The mechanism of action is related to RIF binding to the RNA polymerase near its active center and prevents the initiation of RNA transcription by blocking formation of phosphodiester bond. PVP can act as a capping and etching agent for protection of the outer surface NPs and formation of a mesoporous shell, respectively. Another important feature of this work is the choice of the ligand (1,10-phenanthroline) for precursor Cd complex that works as a chelating agent in order to increase optical and electrical properties and stability of the prepared nanomaterial. The biosensor can significantly enhance electrocatalytic activity towards the oxidation of RIF, under optimal conditions showing high sensitivity and selectivity, good reproducibility and stability and allowing ultra-trace level determination of RIF with very low detection limit (4.55 × 10^−17^ M), and a wide linear range from 1.0 × 10^−16^ to 1.0 × 10^−7^ M in biological and pharmaceutical samples with satisfactory recovery data [90].

**Figure 3:**
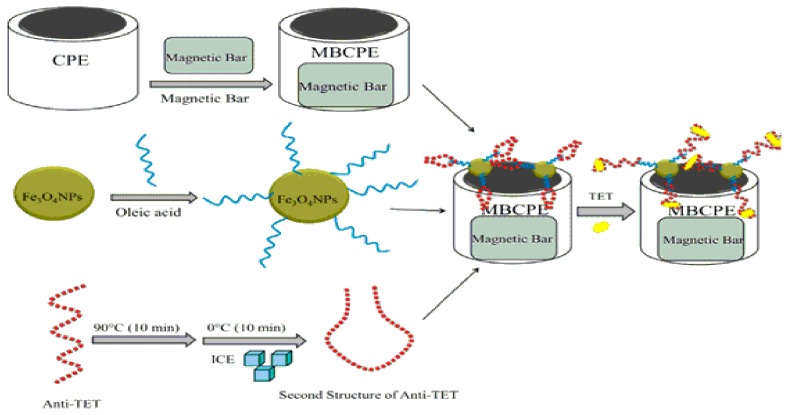
Schematic representation of the proposed electrochemical aptasensor (magnetic bar carbon paste electrode (MBCPE)/Fe_3_O_4_NPs@oleic acid (OA/anti-tetracycline (anti-TET) “Reprinted with permission from [91]. Elsevier and Copyright Clearance Center Inc., 2016”.

MNPs functionalization can be done by several ways: by conjugating targeting ligands (proteins, aptamer), by using different coating materials (Au, Ag, fluorescent molecules etc.), by immobilizing at their surfaces biomolecules, or by mixing them with other nanomaterials (multi wall carbon nanotubes, metallic nanoparticles or biomimetic receptors such as molecular imprinted polymers). An example in which the MNPs were incorporated into the carbon paste electrode was developed for the selective detection of tetracycline (TET) [91]. First a magnetic bar carbon paste electrode (MBCPE) was constructed, and then it was modified with Fe_3_O_4_ MNPs and oleic acid (OA) which possesses the benefit of providing a suitable base for attaching the anti-TET. After immobilization of anti-TET at the MBCPE/Fe_3_O_4_NPs@OA electrode, the proposed aptasensor (MBCPE/Fe_3_O_4_NPs@OA/anti-TET) was fabricated and applied for TET detection in real samples (i.e., tablet drug). It is noticeable that using the synthesized nanomaterial of Fe_3_O_4_NPs and Fe_3_O_4_NPs@OA leads to performing a better immobilization process at the electrode surface. Using the electrochemical impedance spectroscopy (EIS) technique, the linear range and the limit of detection for TET were found to be 1.0 × 10^−14^, 1.0 × 10^−6^ M, and 3.8 × 10^−15^ M, respectively. Using the differential pulse voltammetry (DPV) technique, the linear range and the limit of detection for the mentioned electrode were found to be 1.0 × 10^−12^, 1.0 × 10^−6^ M, and 3.1 × 10^−13^ M, respectively. Some benefits of the mentioned aptasensors are the use of MNPs (e.g., iron oxide), placement of a magnet on the electrode surface which leads to the physical attraction of iron oxide, possessing high selectivity, sensitivity, reproducibility and stability [91]. The schematic representation of the elaboration protocol for the electrochemical aptasenzor is presented in Figure 3.

Kanamycin (KM), an aminoglycoside antibiotic produced by *Streptomyces kanamyceticus*, is generally used in veterinary medicine to inhibit the growth of both Gram-positive and Gram-negative bacteria. The residual amount of KM in food stuff may lead to antibiotic resistance from the pathogenic bacterial strains, which can endanger the consumer [92]. For the sake of consumers’ security, the EU has established maximum residue limits for KM in edible samples: 100 μg kg^−1^ for meat, 600 μg kg^−1^ for liver, 2500 μg kg^−1^ for kidney, and 150 μg kg^−1^ for milk [92]. A novel label-free immunosensor for the sensitive detection of KM based on Ag@Fe_3_O_4_ and thionine—graphene sheets (TH-GS) modified glassy carbon electrode (GCE) was reported [93]. Due to its excellent electron transfer activities and abundant mesoporous structures, Ag@Fe_3_O_4_ could not only improve the electron transfer efficiency but also enhance the immobilizing amount of Abs. Other antibiotics such as gentamicin, streptomycin, and tobramycin can also be captured by their specific Abs to fulfill the detection. The proposed immunosensor shows wide linear range, low detection limit, good reproducibility and selectivity, as well as acceptable stability [93].

**Figure 4:**
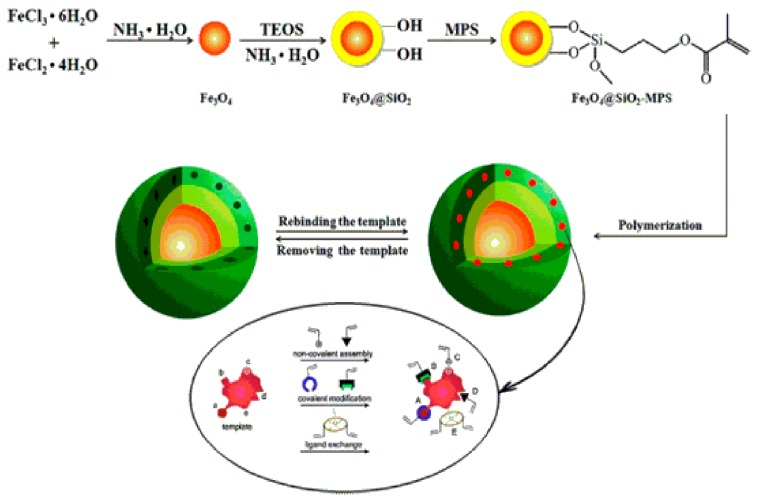
Schematic representation of preparation for magnetic MIPs (MMIPs) “Reprinted with permission from [95]. Elsevier and Copyright Clearance Center Inc., 2017”.

Molecularly imprinted polymer (MIP), a man made predesigned cross-linked structure organic nanomaterial with specific recognition sites complementary in size, shape, and functional groups, with the template molecule, has been developed as a powerful medium to enhance selectivity in electrochemical sensor. Recently, various nanomaterials involving multi-walled carbon nanotubes (MWCNTs), graphene, metal nanoparticles, metal oxides, and semiconductors have been used in the construction of imprinted sensors to improve their analytical performance. MWCNTs have been extensively employed in sensors as modification materials due to their unique structural, large surface area, good physical and chemical stability, and ease of preparation. A magnetic MIP sensor (MMIP-sensor) based on functional of MWCNTs attached Fe_3_O_4_NPs modified electrode was also developed, in which the integration of the MWCNTs and the Fe_3_O_4_ NPs is expected to provide a synergistic effect in electrochemical sensor construction [93,94]. A novel magnetic MIP electrochemical sensor (MMIP-sensor) was developed based on magnetic MWCNTs for the sensitive determination of trace KM in complex matrix (schematically represented in Figure 4) [95]. To improve the sensitivity of the imprinted sensor, the MMIP was prepared based on MWCNTs decorated with Fe_3_O_4_NPs with surface imprinting technique. Moreover, the MWCNTs were grafted with polymethyl methacrylate (PMMA) polymeric film to improve the compatibility, which results in the imprinted layer structure closer and compact. The performance of the MMIP-sensor was investigated by CV and DPV in detail. The results showed that the MMIP-sensor can be used to detect KM with high sensitivity and selectivity. The MMIP-sensor was linearly dependent on KM concentration over the range of 1.0 × 10^−10^–1.0 × 10^−6^ mol L^−1^ with a detection limit of 2.3 × 10^−11^ mol L^−1^. The performances of the MMIP-sensor are due to the Fe_3_O_4_ NPs that enhance electrode conductivity and facilitate electron transfer allowing the KM detection in complex real samples [95].

Another hybrid material based on MWCNT decorated with synthesized NiFe_2_O_4_ MNPs was used for sensor design [96]. This composite was used as a mediator for the voltammetric determination of cefixime, an oral third generation cephalosporin antibiotic. Cefixime is used to treat infections caused by bacteria such as pneumonia, bronchitis, gonorrhea, ear, lung, throat and urinary tract infections. Forty to fifty percent of the oral dose of cefixime is adsorbed from the gastrointestinal tract. To evaluate the applicability of the proposed sensor, cefixime was determined in tablet, blood plasma, and urine samples using standard addition method. Under the optimum conditions, two wide linear dynamic ranges of 0.1–100 and 100–600 µmol L^−1^ and a detection limit of 0.02 µmol L^−1^ was found for cefixime [96].

Comparing the results obtained on different experimental configurations, it can be observed that the analytical performances of the sensors are influenced by the nature and by the properties of the surface modifiers. In each case the functionalization of the electrode determined the enhancement of the selectivity towards the analyte (different antibiotics). The use of MNPs and MNPs composite based materials in sensing devices apparently improves the results obtained in their absence mainly due to the electronic and electrocatalytic properties. Moreover, MNPs present enhanced versatility, and can be functionalized in different manners in order to provide synergistic effects in electrochemical sensors and biosensors that can boost their common properties.

The performances of all the configurations were tested on real samples such as commercial drugs, human serum, urine, as well as food and soil samples. A mandatory condition for the determination of antibiotics in a complex matrix is represented by the high selectivity of the sensor towards these analytes, as the tests are performed in the presence of possible interferents or different metabolites. The presence of nonfunctionalized or functionalized MNPs usually allows the detection of the target analyte in real samples without the need of separation.

### 4.2. Optical Sensors for Antibiotics

Generally, optical-magnetic bead based biosensors exhibit excellent optical performance because of the unique interactions between light waves and the surface coating materials such as Au, Ag, QDs and fluorescent molecules, displaying excellent localized surface plasmon resonance (SPR), surface-enhanced Raman scattering (SERS), and fluorescence. Fiber optic biosensors are optical biosensors with working principle based on the reflective properties of light to sense numerous analytes simultaneously [97]. A high sensitivity fiber optic biosensor based on the localized surface plasmon coupled fluorescence (LSPCF) was demonstrated by Hsieh et al. [98]. The metallic nanoparticles provide a strong local field to enhance the fluorescence signal of fluorophores. They also help to scatter the fluorescence signal, and to increase the far field detectable signals of the fiber optic biosensor.

Localized surface plasmon resonance (LSPR) is a powerful technique for chemical and biological sensing experiments. It provides a solution with reasonably affordable cost (due to the simplicity of instrumentation) for portable devices [99] with fast, reproducible, small sample volumes, high sensitivity and selectivity and label-free sensing technique. LSPR has been shown to be an effective platform for the detection of biomolecules with chemical relevance [100]. It also offers the advantage of being easily multiplexed to enable high throughput screening in an array format for simultaneous detection [101,102,103], high sensitivity to the refractive index change of the analyte [104], a small footprint [105] and low cost instrumentation [106]. The LSPR is responsible also for the electromagnetic field enhancement that leads to surface enhanced Raman scattering (SERS measurements) for the detection of biomolecules [107,108,109,110].

SPR differs from LSPR as the induced plasmons oscillate along the metal-dielectric interface rather than locally to the nanostructure [111,112]. The decay length of the electromagnetic field observed is in the order of 200 nm for surface plasmon and 6 nm for localized surface plasmons (both decaying exponentially). There are two main sensing modes that can be implemented:

- *Wavelength-shift sensing*, is used to monitor resonant wavelengths of light with the refractive index changes determined by the presence of analyte at the surface. The wavelengths of light cause the collective oscillation of valence electrons and subsequent absorption within the ultraviolet-visible (UV-Vis) band, due to interactions between the incident photons and the conduction band of a noble metal nanostructure.
- *Surface-enhanced techniques*, offer the detection of a target analyte by monitoring changes in the enhanced electromagnetic fields. Maximum enhancement is obtained when the LSPR wavelength is situated between the excitation wavelength and the wavelength of the scattered photon. This dependence of SERS on LSPR wavelength is complementary for molecular binding and identification studies.

Several elements have been shown to support localized surface plasmons: structural factors of the metallic nanostructures such as dimension, shape and spacing [113,114], metal composition (palladium (Pd), platinum (Pt), gold (Au) and silver (Ag)), and diameter of the nanostructure (larger diameters also have the effect of producing broader spectra) [115,116]. Optical geometries that are employed within LSPR sensors: transmission [117], reflection [118], dark field scattering [119] and total internal reflection [120].

Several antibiotic detection devices based on SPR are presented in literature for vancomycin [121,122], chloramphenicol [123], different types of fluoroquinolones (FQ) [124], and ciprofloxacin [125]. Ampiciline detection was performed using an SPR sandwich based biosensor [126]. An alternative optical technique based on Laser Doppler Micro-Electrophoresis with UV-Vis spectroscopy was used for gentamicin detection [127]. The application of magnetic and core shell nanoparticles to determine enrofloxacin was mentioned in Kim et al. [128] by using a laser induced fluorescence microscope based technique. An innovative wavelength interrogated optical system (WIOS)], which is based on a waveguide grating and wavelength modulation was reported by Adrian et al. [129]. More than 25 antibiotics can be detected by using a typical planar microarray configuration based on WIOS [130].

Cantilever optical sensors have been recognized recently as a promising nanomechanical sensor platform based on nanoparticles, for various chemical and biological applications [131,132,133]. The optical cantilever device is based on the principle of variable light coupling. The amount of light coupled from the input to the output waveguide changes as the cantilever moves up or down. Changes in the light intensity serves for the indirect measurement of the cantilever displacement on the sensing surface [134,135].

**Figure 5:**
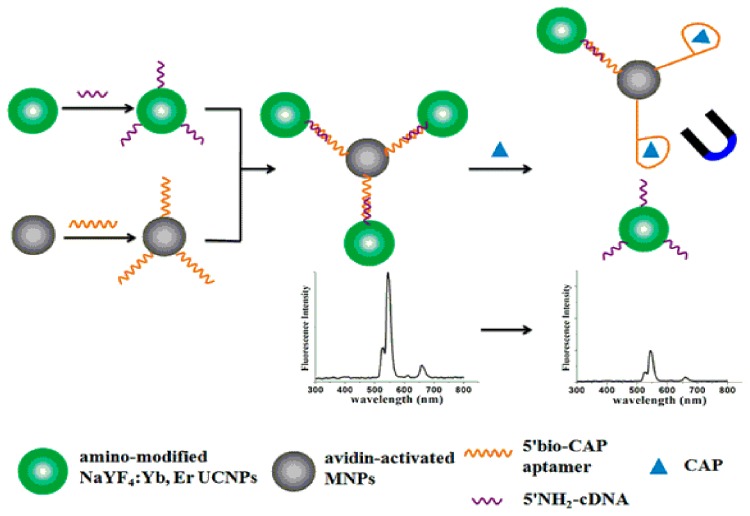
Schematic representation of the fluorescent nanoprobe-based bioassay for the determination of chloramphenicol (CAP) “Reprinted with permission from [80]. Elsevier and Copyright Clearance Center Inc., 2015”.

Wu et al. [80] proposed a fluorescence-based aptasensor described in Figure 5, using aptamer conjugated MNPs for chloramphenicol (CAP) recognition and concentration evaluation. In the absence of the target molecules, MNP-aptamer complex hybridizes to its complementary DNA (cDNA) modified with up conversion nanoparticles (UCNPs) to form the duplex structure giving a maximum fluorescent signal. Upon CAP addition, the aptamer preferentially binds with CAP and causes the dissociation of some cDNA, liberating some UCNPscDNA complexes, and leads to a decreased fluorescence signal on the surface of MNPs. Under optimal conditions, a linear CAP detection range from 0.01 to 1 μg L^−1^ was achieved.

CAP was detected by a facile fluorescence “switch-on” scheme in food, based on a novel magnetic aptamer probe (aptamer-Pt-luminol nanocomposite labeled with hemin/G-quadruplex). The composite probe was firstly prepared through the immuno-reactions between the capture beads (anti-dsDNA antibody labeled on magnetic Dynabeads) and the nanotracer (nano-Pt-luminol labeled with double-strand aptamer, as ds-Apt, and hemin/G-quadruplex). When the composite probe is mixed with CAP, the aptamer preferentially reacted with CAP to decompose the double-strand aptamer to ssDNA, which cannot be recognized by the anti-dsDNA antibody on the capture probes. Thus, after magnetic separation, the nanotracer was released into the supernatant. Because the hem in/G-quadruplex and PtNPs in nanotracer can catalyze luminol-H_2_O_2_ system to emit fluorescence, a dual-amplified “switch-on” signal appeared, of which intensity was proportional to the concentration of CAP between 0.001 and 100 ng mL^−1^ with a detection limit of 0.0005 ng mL^−1^. The sensor was used for CAP detection in real milk samples [82].

Colorimetric sensors for antibiotics were also proposed due to their simplicity in use and visible detection, by naked eye. Tetracyclines (TET) are known to have a strong tendency to complex with metal ions such as Fe(II) and Fe(III) and this could be done on the surface of Fe_3_O_4_MNPs. Knowing, this, a novel colorimetric biosensor for the determination of TET based on the Fe_3_O_4_ magnetic nanoparticles (Fe_3_O_4_MNPs) was reported [136]. The complexation of Fe_3_O_4_MNPs and TET could lead to the accelerated catalysing H_2_O_2_-mediated oxidation of 3,3′,5,5′-tetramethylbenzidine (TMB) by Fenton chemistry. The reaction starts with oxidation of Fe_3_O_4_MNPs-TET complex by dissolved oxygen to generate reactive oxygen species (ROS), primarily-OH, leading to deepen the color of the solution. Analysis results can be seen with the naked eye and monitored by UV-Vis spectra. Under optimal conditions, the detection limits were 26 nM for oxytetracycline (OTC), 45 nM for TET and 48 nM for doxycycline (DOX). Compared with other analytical techniques, the proposed colorimetric biosensor is advantageous in terms of convenient operation without any complicated chemical synthesis, modification, or tedious experimental procedures and its simple signal generation and detection [136].

## 5. Conclusions

Nowadays, the environment is constantly exposed to an extensive mixture of antibiotics as well as other drugs, which affect the quality of water, soil, and food influencing human health. The increasing demands for monitoring systems capable of routine and high throughput analyses is a hot topic for interdisciplinary researches.

In their native form, magnetic nanoparticles are not target-selective, being thus unsuitable for application in complex matrices. Such inconvenient situation can be overcome by suitable functionalization or coating. Chemical or physical modification with organic compounds such as chelating agents determine the increase selectivity for different metal ions with application in heavy metal detection and removal. The use of magnetic nanoparticles for the elaboration of sensors for the detection of antibiotics has been extensively reported. Special care should be considered in aquatic environments where high concentrations of other pollutants are found (heavy metals, salt ions, and organic compounds), or in waste waters. Several separation techniques were developed and are still in use for the detection of antibiotics in such media to overcome the influence of these interfering species on the signal of the analytes. For example, heavy metals are reducing agents for magnetic nanoparticles and their presence in real samples together with the antibiotics can hinder the target detection. In these situations the use of electrochemical and optical sensors based on magnetic nanoparticles functionalized with molecularly imprinted polymers and/or on antibody/aptamer formats could represent a better choice. Nevertheless, these devices provide a fast response, can be miniaturized, and they also possess a good tolerance against various matrix effects, while the improvement of the sensitivity is improved due to the use of nanomaterials.

The diversity of antibiotics classes released in the environment by farming and agricultural activities required sensors with a high degree of selectivity and sensitivity, as well as a high capability for in-field analysis. Developments in the miniaturization of sensors and actuators, improvements in micromachining technologies, microelectronics, micro fluidics and packaging, together with developments in aptamers and other biomimetic molecules, have led to new generation of “smart” sensors that are also effective and cheap, and can be applied for the detection of antibiotics in various matrices. By using simple and suitable approaches, the detection of antibiotics could provide important background data for risk assessment, and better contamination control of these emergent pollutants.

Facing a real menace due to the increased resistance to the widely used antibiotics, researchers must provide fast and sensitive methods and devices that would allow the detection and quantification of small amount of various molecules from complicated matrices such as sewage waters, waste water, soil, and food samples. Electrochemical and optical sensors assisted by several nanomaterials could definitely meet this challenge. Due to their versatility and multiple possibilities of functionalization, MNPs are promising components, providing anchoring platform for biomolecules, allowing an improvement in sensitivity and selectivity. When designing electrochemical sensors, one should consider the increased active surface on which a large number of biomolecules could be attached. MNPs could assure an increase of sensitivity as well as selectivity, as their large surface area provides a higher coverage on the electrode surface, and an increased number of biomolecules (Abs, aptamers). The ideal sensor for antibiotics detection will have the sensitivity of optical sensors and the selectivity of electrochemical sensors. The drawbacks of optical and electrochemical sensors could be avoided by simultaneous use of the two types of transducers modified with MNPs. in hybrid approaches. There are a number of issues to be tackled by biosensor technology before they become a routine technique for antibiotics detection. New technology such as 3D printing, bioengineering and microengineering, micromotors, and artificial intelligence represents outstanding tools that, in the near future, will be part of biosensors and will transform them as a method for antibiotic monitoring.

## Figures and Tables

**Table 1:** Different routes for magnetic nanoparticles synthesis.

Synthesis Method	Advantages	Disadvantages
**Co-precipitation**	- most proper method for the synthesis of magnetic nanoparticles (MNPs) of controlled sizes and magnetic properties; - extensively used for biomedical applications due to ease of application, and less need for harmful materials and procedures	- produces particles with extensive distribution of particle size, which sometimes requires secondary size selection; - the size of MNPs decreased with increasing pH value and ionic strength in the medium; - the tendency of the particles to agglomerate because of extremely small particle size, leading to greater specific surface area and high surface energy
**Thermal and hydrothermal decomposition**	- reactions are performed in aqueous media in reactors or autoclaves where the pressure can be higher than 2000 psi and the temperature can be above 200 °C; - the reaction conditions (e.g., solvent, temperature, time, etc.), usually have important effects on the products	- laborious purification steps are needed before the end product can be used in biomedical applications; - the production of organic-soluble nanoparticles (NPs) limit the range of applications in biological fields; - surface treatment is needed after synthesis; - the resulting NPs are generally dissolved in nonpolar solvents
**Microemulsion and inverse micelles**	- the use of simple equipment; - the possibility of synthesizing a great variety of materials with a high degree of control over particle size and composition; - the preparation of NPs with crystalline structure and high specific surface area using simple conditions of synthesis (near ambient temperature and pressure); - the particles obtained with these methods are smaller in size but higher in saturation magnetization	- the challenges in their scale up procedures; - the side effects of the remaining surfactants on the properties of the particles
**Sol-gel processes**	- the main parameters that influence the kinetics, growth reactions, hydrolysis, condensation reactions, and consequently, the structure and properties of the gel (e.g., solvent nature, temperature, concentration of the salt precursors pH, stirring, etc.) are easy controlled; - the preparation of pure amorphous phases, with monodispersity and good control of the particle size, as well as a predetermined structure is possible only by adjusting the experimental conditions; - the microstructure and the homogeneity of the reaction products are controllable	- these reactions are performed at room temperature, further heat treatments are needed to acquire the final crystalline state; - the by-products generated from reactions are pollutants; - the need for post-treatment of the products; - tree dimension (3D) oxide networks are produced, thus limiting the efficiency
**Biosynthesis**	- green and ecofriendly method using bacteria and other microorganisms	- the necessity of strict anaerobic conditions
**Electrochemical methods**	- allow the creations of 3–8 nm maghemite particles from an iron electrode in an aqueous solution of dimethylformamide DMF and cationic surfactants; - only the adjustment of the current density controls the particle size	- limited use due to the specific experimental conditions [54,55]
**Flow injection synthesis**	- high reproducibility because of the plug-flow and laminar conditions; - high mixing homogeneity and opportunity for a precise external control of the process	- magnetite NPs had a narrow size distribution in the range of 2–7 nm
**Aerosol/Vapor methods (spray and laser pyrolysis)**	- allow high rate production; - MNPs with size ranging from 5 to 60 nm with different shapes have been obtained using different iron precursor salts in alcoholic solution; - MNPs have higher crystalline structure and saturation magnetization	
**Coatings and functionalization at the surface of NPs**	- tuning the overall properties of particles to fit targeted applications	- the need for organic or inorganic materials involving supplementary steps

**Table 2:** The most widely used classes of antibiotics and their application/side effects “Reproduced (adapted) with permission from [59]. American Chemical Society, 2015”.

Antibiotics	Applications	Side Effects
**Penicillins**	- wide range of infections with staphylococci and streptococci	- rashes - fever - allergic reactions
**Tetracyclines**	- infections of respiratory tract; - infections of urinary tract; - extensively used in veterinary and aquaculture medicine	- hepatotoxic
**Sulfonamides**	- active against a broad spectrum of gram-positive and gram-negative bacteria, *Plasmodium* and *Toxoplasma* sp.; - infections of urinary tract	- diarrhea - nausea - vomiting - serious skin rushes
**Macrolides**	- streptococcal and pneumococcal infections; - infections of respiratory tract; - feed additive for animals; - drugs of choice for infections due to *Mycoplasma pneumoniae*, *Legionella* sp., *Bordetella pertussis* or *Corynebacterium diphtheriae*	- gastrointestinal disturbances
**Fluoroquinolones**	- infections of genitourinary; - infections of respiratory tract; - skin infections	- severe hepatic toxicity - hemolytic anemia - coagulopaty - tendonitis and tendon rupture
**Aminoglycosides**	- infections from aerobic and Gram-negative bacteria	- affects kidney, liver and ear functions
**Phenicol**	- veterinary use	- anemia
**Licosamides**	- veterinary use	- gastrointestinal problems

**Table 3:** Analytical methods based on MNPs used for antibiotics detection.

Method/Application	Analytical Parameters	Observations	Reference
**Liquid chromatography (LC)-based coupled methods**			
**HPLC-UV/Determination of ceftriaxone in human plasma** **(HPLC-UV—high performance liquid chromatography with UV detector; LR—linear range; LOD—lowest limit of detection)**	LR: 0.06–40 µg mL^−1^ LOD: 20 ng mL^−1^	Ag modified- magnetic nanoparticle (Ag-MNPs) were used for the preconcentration of ceftriaxone, an enrichment factor of 19 being obtained in optimal conditions	[72]
**HPLC-UV/Determination of chloramphenicol (CAP), florfenicol (FF) and thiamphenicol (TAP) in water, chicken blood and egg samples**	LOD: CAP: 0.16 µg kg^−1^ FF: 0.08 µg kg^−1^ TAP: 0.08 µg kg^−1^ Recoveries from 88.3% to 99.1%	A magnetic mesoporous dual-template molecularly imprinted polymer (Fe_3_O_4_@mSiO_2_@DMIP) was synthetised;-The obtained Fe_3_O_4_@mSiO_2_@DMIP particles were applied as a magnetic solid-phase extraction sorbent for the rapid and selective extraction of CAP, FF, and TAP	[73]
**LC-MS/MS/Determination of tetracycline, chlortetracycline, oxytetracycline and tyosin in a field fertilized with liquid manure** **(LC-MS/MS—LC with tandem mass spectrometry)**	Amount detected: Tetracycline from 86.2 to 4000 µg kg^−1^ Chlortetracycline from 4.6 to 100 µg kg^−1^ Oxytetracycline not detected Tyosin not detected	Tetracyclines were detected in the environment, are persistent residues, and accumulate in soil	[74]
**HPLC-MS/MS/Determination of erythromycin, tylosin and other antibiotics from surface waters, soil and liquid manures**	Amount detected:Erythromycin in water 0.3 µg L^−1^ Sulfadiimide in soil 1000-2000 µg kg^−1^	Test were performed seven months after application, this indicates the high stability of some antibiotics in manure and soil	[75]
**UHPL-DAD/Determination of 11 sulfonamide antibiotics in mineral waters with different mineral content (UHPL-DAD—ultra-high pressure liquid chromatography-diode array** **Detection)**	LODs lower than 32 pg mL^−1^ for all of the analyzed compounds	Pristine multi-walled carbon nanotubes (MWCNTs) and magnetic-MWCNTs (m-MWCNTs) were used as sorbents for off-line dispersive solid-phase extraction (dSPE) of antibiotics from mineral waters	[76]
**Electrochemical sensors**			
**EIS/Determination of tetracycline in milk**	LR: 0.08–1 ng mL^−1^ LOD: 0.032 ng mL^−1^	Electrochemical immunosensor based on gold electrode-modified carboxyl-Fe_3_O_4_ nanoparticle (MNPs) by chitosan (CS) as linker	[77]
**EIS/Determination of penicillin in milk**	LR: 0.1–200 ng mL^−1^ LOD: 0.057 ng mL^−1^	Electrochemical aptasensor based on GCE modified with poly(3,4-ethylenedioxythiophene)–gold nanoparticles composite (PEDOT–AuNPs) and magnetic graphene nanocomposite (GR–Fe_3_O_4_NPs)	[78]
**Voltammetry/Determination of chloramphenicol in fish samples**	LR: 0.001–100 ng mL^−1^ LOD: 0.33 pg mL^−1^	Voltammetric aptasensor based on magnetic gold nanoparticles (Fe_3_O_4_@Au) and a dendritic polymerase used in order to link these nanocomposite to quantum dots (CdS or PbS) and to form the nanotracers	[79]
**Optical sensors**			
**Fluorescence biosensor/Determination of chloramphenicol in milk**	LR: 0.01–1 ng mL^−1^LOD: 0.01 ng mL^−1^	Aptasensor based on aptamer-conjugated magnetic nanoparticles (MNPs)	[80]
**Chemiluminescent biosensor/Determination of chloramphenicol in milk**	LR: 0.01–0.20 ng mL^−1^ LOD: 0.01 ng mL^−1^	Aptasensor based onmagnetic nanoparticles (MNPs) and thiolated hybridized complementary strand modified *N*-(4-aminobutyl)-*N*-ethylisoluminol (ABEI)-functionalized flower-like gold nanostructures (AuNFs)	[81]
**Colorimetric biosensor/Determination of oxytetracycline (OTC), tetracycline (TC) and doxycyline (DOX)**	OTC: LR: 50–1000 nM LOD: 26 nM TC: LR: 100–1000 nMLOD: 45 nM DOX: LR: 50–1000 nM LOD: 48 nM	Enzyme sensor based on Fe_3_O_4_ magnetic nanoparticles (Fe_3_O_4_ MNPs) The elaborated biosensor was applied for the determination of DOX content in drugs	[82]
